# The Influence of Age and Gender on Forehead Soft Tissue Thickness: A Magnetic Resonance Imaging-Based Study

**DOI:** 10.1007/s00266-025-04957-y

**Published:** 2025-06-04

**Authors:** Cagri Cakmakoglu, Grzegorz Kwiecien, Layne N. Raborn, James R. Gatherwright, Addison Barnett, Marjorie Kragel, Pierce L. Janssen, James E. Zins

**Affiliations:** 1 Department of Plastic Surgery, Cleveland Clinic, Cleveland Clinic Lerner College of Medicine, 9500 Euclid Avenue, A60, Cleveland, OH 44195 USA; 2 Division of Plastic Surgery, Cleveland Clinic, Akron General Hospital, Akron, Ohio USA; 3 Division of Plastic and Reconstructive Surgery, University of Rochester Medical Center, Rochester, NY USA

**Keywords:** Photoaging, Gender, Forehead thickness, Skin thickness

## Abstract

**Background:**

Variations in soft tissue thickness related to age and gender are clinically significant for addressing facial aging and planning esthetic interventions. Previous studies have assessed skin thickness using various methods, including ultrasonography and biopsies, but data specifically examining forehead soft tissue thickness by age and gender are limited.

**Objective:**

This study aimed to investigate the effects of age and gender on forehead soft tissue thickness using MRI, a non-invasive modality that provides excellent contrast resolution and reliable measurements.

**Methods:**

We conducted a retrospective analysis of 160 Caucasian adults (80 females, 80 males) aged 20–99 years, grouped by decade. We measured midline and lateral forehead soft tissue thickness in the sagittal plane on T1-weighted MRI sections, from skin to external cortical lamina, in the lower, middle, and upper thirds. Statistical analyses were performed using factorial ANOVA to assess the impact of age and gender.

**Results:**

Males had significantly greater forehead soft tissue thickness than females at all ages and across most regions. Notable differences were observed in the midline (5.4 mm in males vs. 5.0 mm in females; *p* = 0.043) and in the lower third of the lateral forehead (9.4 mm in males vs. 8.6 mm in females; *p* = 0.008). Thickness increased from the third to the fifth decade, peaking between ages 40–49, then declining in later decades, particularly in the lower third.

**Conclusion:**

Forehead soft tissue thickness varies significantly by age and gender, highlighting the importance of considering these differences in planning esthetic procedures for optimal outcomes.

**Level of Evidence IV:**

This journal requires that authors assign a level of evidence to each article. For a full description of these Evidence-Based Medicine ratings, please refer to the Table of Contents or the online Instructions to Authors www.springer.com/00266.

## Introduction

Skin, the primary interface between the human body and the external environment, is subject to both intrinsic and extrinsic processes that alter its structure and function with time [1, 2]. At a microscopic level, aging causes histological changes that increase skin fragility and risk of damage [2]. It also contributes to skin elastosis, epidermal atrophy, and collagen fragmentation. Fair skin types exhibit a higher degree of actinic damage compared to darker skin types under similar actinic environmental exposure [2–5]. Macroscopically, aging manifests as rhytid formation, loss of skin elasticity, xerosis, dyspigmentation, soft tissue deflation, and changes in skin thickness.

Prior studies have examined the relationships between age and gender on skin soft tissue thickness [6–34]. The few studies that specifically examined differences in forehead soft tissue thickness with regard to age and gender have yielded conflicting results and have been inconsistent regarding location studied and methodology. Most of these prior studies have employed ultrasound or cadaver biopsies, which both have their own limitations. Furthermore, a major deficiency in the literature is a lack of age-stratified analysis of forehead tissue thickness in live subjects, with many studies grouping subjects into “young” and “old” categories rather than evaluating trends over the decades [11, 24, 27, 30–32]. Given the popularity of both surgical and minimally invasive cosmetic procedures in patients over 50, there is likely value in understanding how the face ages from decade to decade over the entire spectrum of adulthood [35]. This study aims to investigate the relationship between age and gender on forehead soft tissue thickness using magnetic resonance imaging (MRI), a widely available and non-invasive modality that offers superior contrast resolution and more reliable thickness measurement when compared to ultrasonography.

## Methods

Institutional review board approval was obtained for this study (Protocol #14-525). A retrospective cohort of Caucasian adult subjects’ MRI head images identified by CPT code was obtained. MRI examinations were performed in the supine position with a 3T MRI scanner (Siemens Healthineers, Erlangen, Germany) using 3 mm or 4 mm layer thickness. All subjects had normal MRI findings on documented radiological evaluation. Individuals with previous facial or head soft tissue injuries, bone injuries, or irregularities identified on imaging were excluded. Subjects were categorized into groups based on age and gender. Ten male and 10 female subjects were included for each of the following age groups: 20–29, 30–39, 40–49, 50–59, 60–69, 70–79, 80–89, and 90–99 years old. This represented a sample of convenience.

**Fig. 1:**
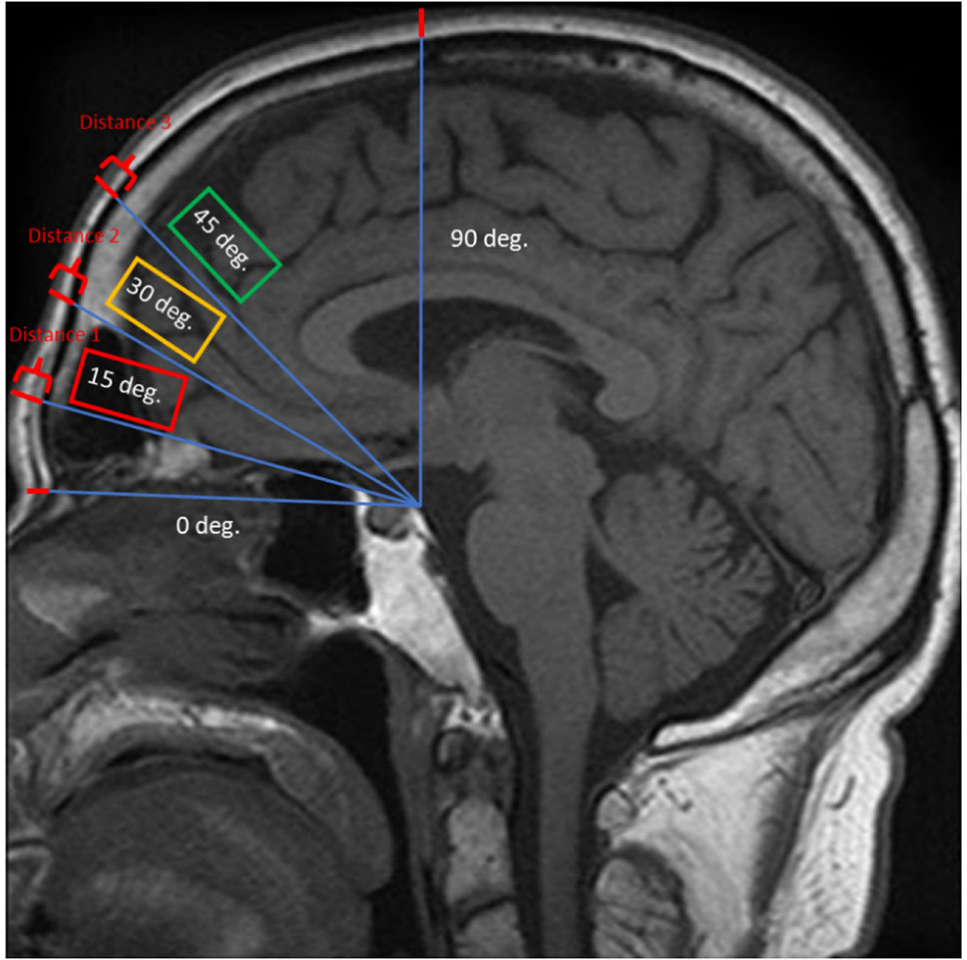
Example of the analyzed midline sagittal plane imaging cut. Lines were drawn from the posterior clinoid process to the outer cortical lamella of the frontal bone at angles of 15-, 30-, and 45-degree. The distance from the epidermal skin surface to the external cortical lamina was measured at these angles, representing soft tissue thickness

T1-weighted MRI images were analyzed for each subject in each of the above groups. Sagittal plane cuts at the right and left supraorbital foramen and midline were utilized to ensure repeatable and consistent soft tissue thickness measurements by a single plastic surgery resident physician. Four lines were drawn on each sagittal cut from the posterior clinoid process to the outer cortical lamella of the frontal bone at 0-degree, 15-degree, 30-degree, 45-degree, and 90-degree angles. Distance from the epidermal skin surface to the external cortical lamina was measured at angles 15- (lower forehead), 30- (middle forehead), and 45-degree (upper forehead) by a single author based on previous methodology used by Frank et al. [36] (Fig. 1).

Statistical analysis was performed using SPSS Statistics 23 (IBM, Armonk, NY). A factorial ANOVA was conducted to compare the gender and age effects on forehead soft tissue thickness. Statistical significance was defined as *p* < 0.05.

## Results

A total of 160 patients were included in this study (80 males and 80 females). MRI measurements were collected, yielding 148–160 measurements for each area of the forehead. The average measured soft tissue thickness ranged from 4.9 ± 1.5 mm to 9.3 ± 2.2 mm.

### Gender and Forehead Soft Tissue Thickness

**Fig. 2:**
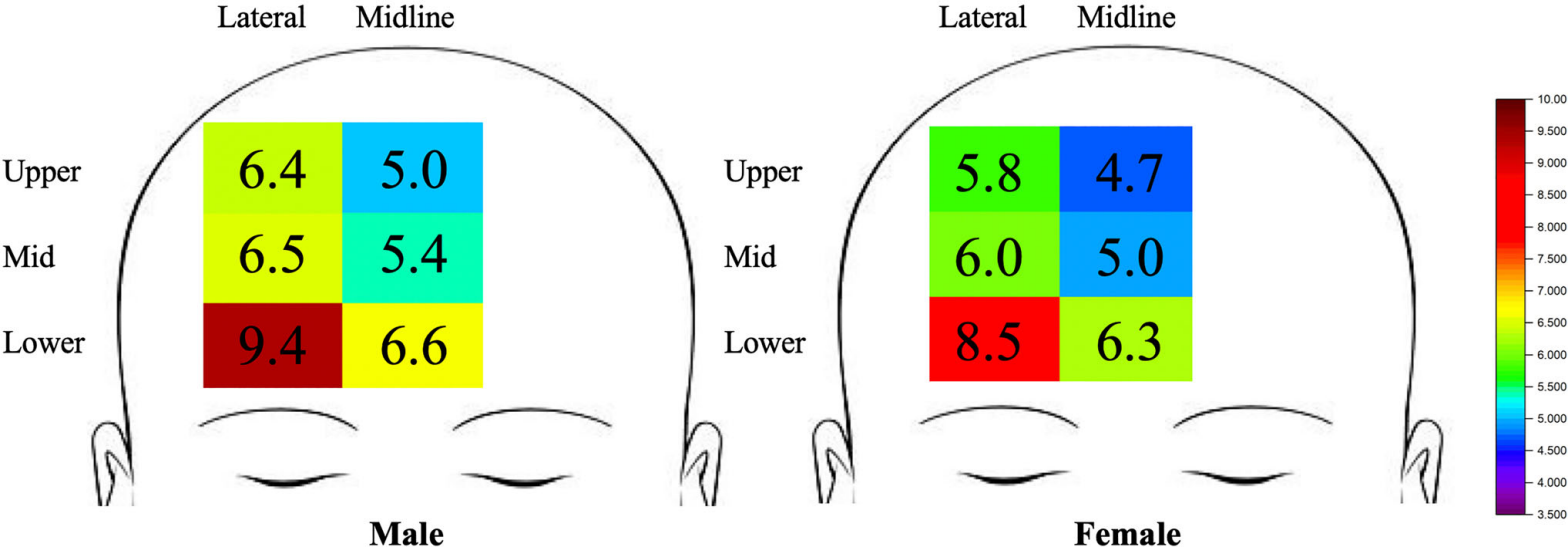
Average forehead soft tissue thickness measurements (in mm) for males and females. Data are expressed as a heatmap, with warmer colors indicating thicker tissue and cooler colors indicating thinner tissue. This Fig. demonstrates that for both genders, soft tissue thickness was greatest in the lower lateral location (red) and thinnest in the upper midline location (blue)

Forehead soft tissue thickness measurements at six different locations (upper, middle, and lower thirds in the midline; upper, middle, and lower thirds laterally) compared to gender are shown in Table 1 and Fig. 2. Figure 2 demonstrates the data as a heatmap, with warmer colors (reds) indicating thicker tissue and cooler colors (blues) indicating thinner tissue. Soft tissue thickness differed significantly between males and females in the middle third of the midline forehead (5.4 vs. 5.0 mm, respectively; *p* = 0.043). There were no statistically significant differences between males and females in the upper or lower thirds of the midline forehead. Soft tissue thickness differed significantly between males and females in all thirds of the lateral forehead. The most significant difference was noted in the lower third of the lateral forehead (9.4 vs. 8.6 mm, respectively; *p* = 0.008), followed by the upper third of the lateral forehead (6.4 vs. 5.8 mm, respectively; p = 0.018) and middle third of the lateral forehead (6.5 vs. 6.0 mm, respectively; *p* = 0.04) (Figs. 5 and 6). Average soft tissue measurements were significantly larger for males compared to females in the lateral forehead (7.4 vs. 6.8 mm, respectively; *p* = 0.007).

**Table 1:** Average soft tissue thickness at various forehead locations stratified by gender

Forehead location		Forehead soft tissue thickness (mm)		
		Male	Female	*p*-value
Midline	Upper 1/3^rd^	5.0	4.7	0.139
	Middle 1/3^rd^	5.4	5.0	**0.043**
	Lower 1/3^rd^	6.6	6.3	0.187
	Average	5.7	5.3	0.08
Lateral	Upper 1/3^rd^	6.4	6.4	**0.018**
	Middle 1/3^rd^	6.5	6.0	**0.044**
	Lower 1/3^rd^	9.4	8.5	**0.008**
	Average	7.4	6.8	**0.007**

Bold vaues indicate *p* < 0.05

### Age and Forehead Soft Tissue Thickness

**Fig. 3:**
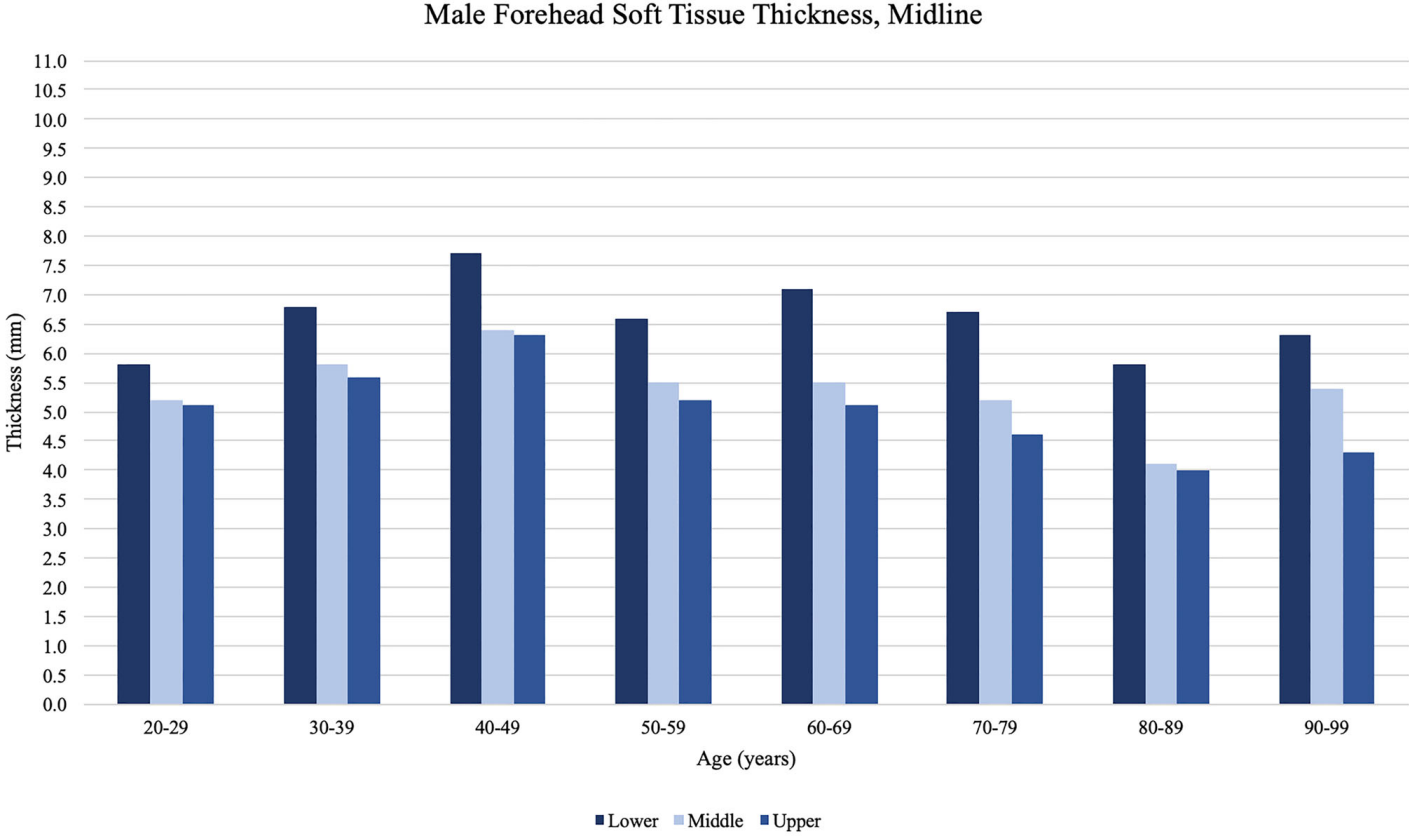
Midline forehead soft tissue thickness measurements obtained from male participants, organized by the age of participant at the time of imaging. Peak thickness is reached by the fifth decade of life and subsequently decreases with age. At the midline forehead, males showed thicker measurements at the lower third and thinner measurements at the upper third

**Fig. 4:**
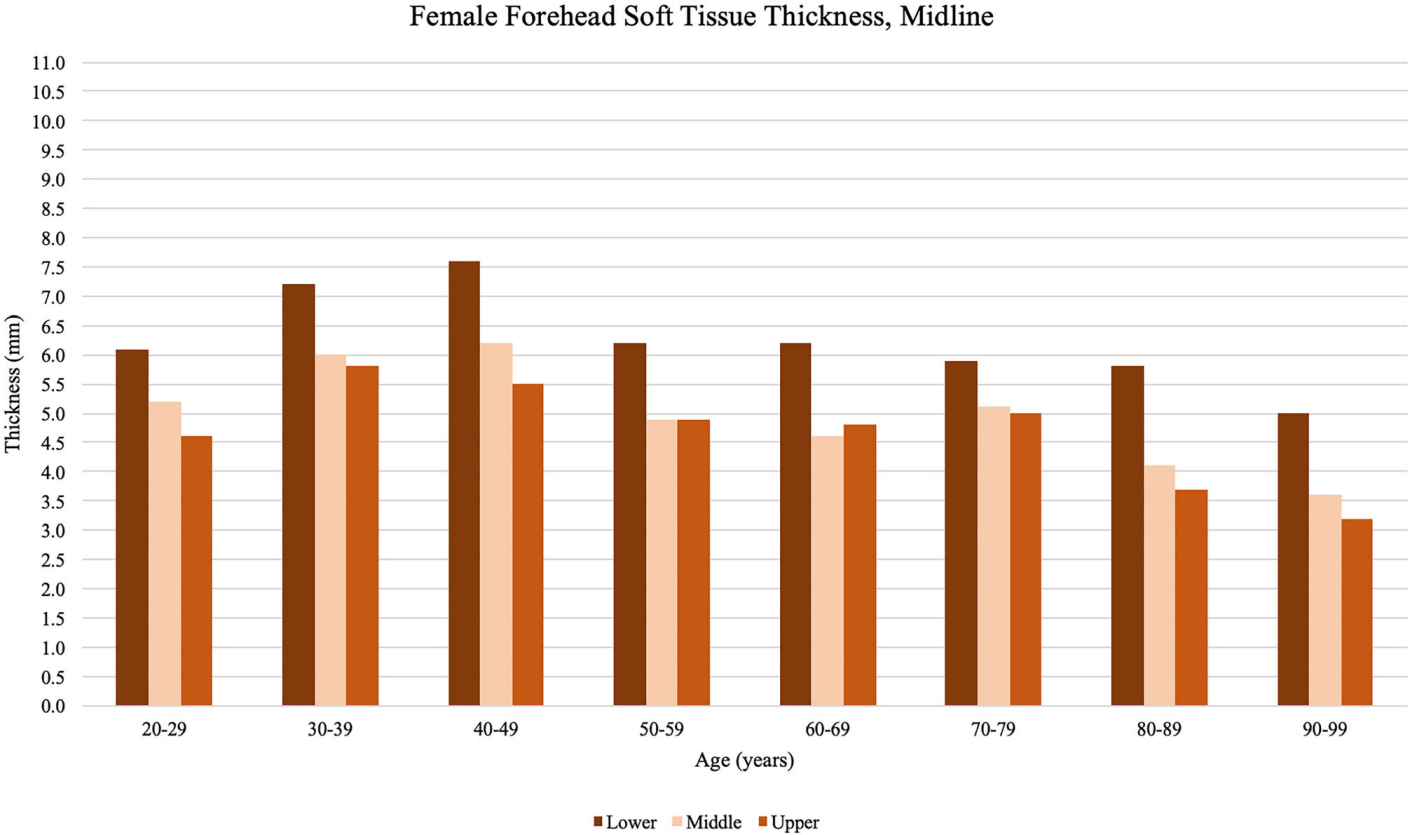
Midline forehead soft tissue thickness measurements obtained from female participants, organized by the age of participant at the time of imaging. Peak thickness is reached by the fifth decade of life and subsequently decreases with age. At the midline forehead, females showed thicker measurements at the lower third and thinner measurements at the upper third with some variability by age

**Fig. 5:**
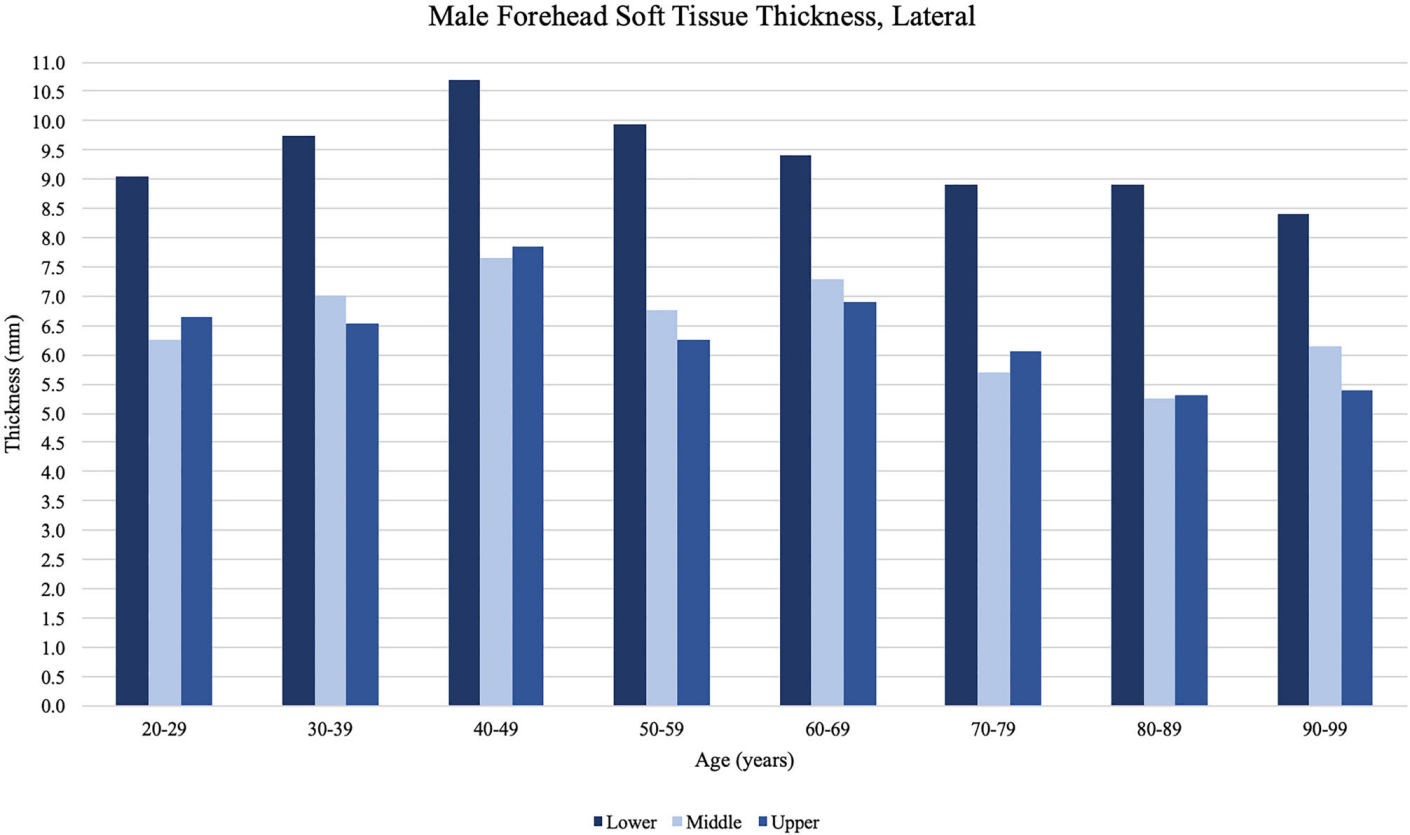
Lateral forehead soft tissue thickness measurements obtained from male participants, organized by the age of participant at the time of imaging. Peak thickness is reached by the fifth decade of life and subsequently decreases with age. At the lateral forehead, males showed thicker measurements at the lower third compared to the middle and upper thirds

**Fig. 6:**
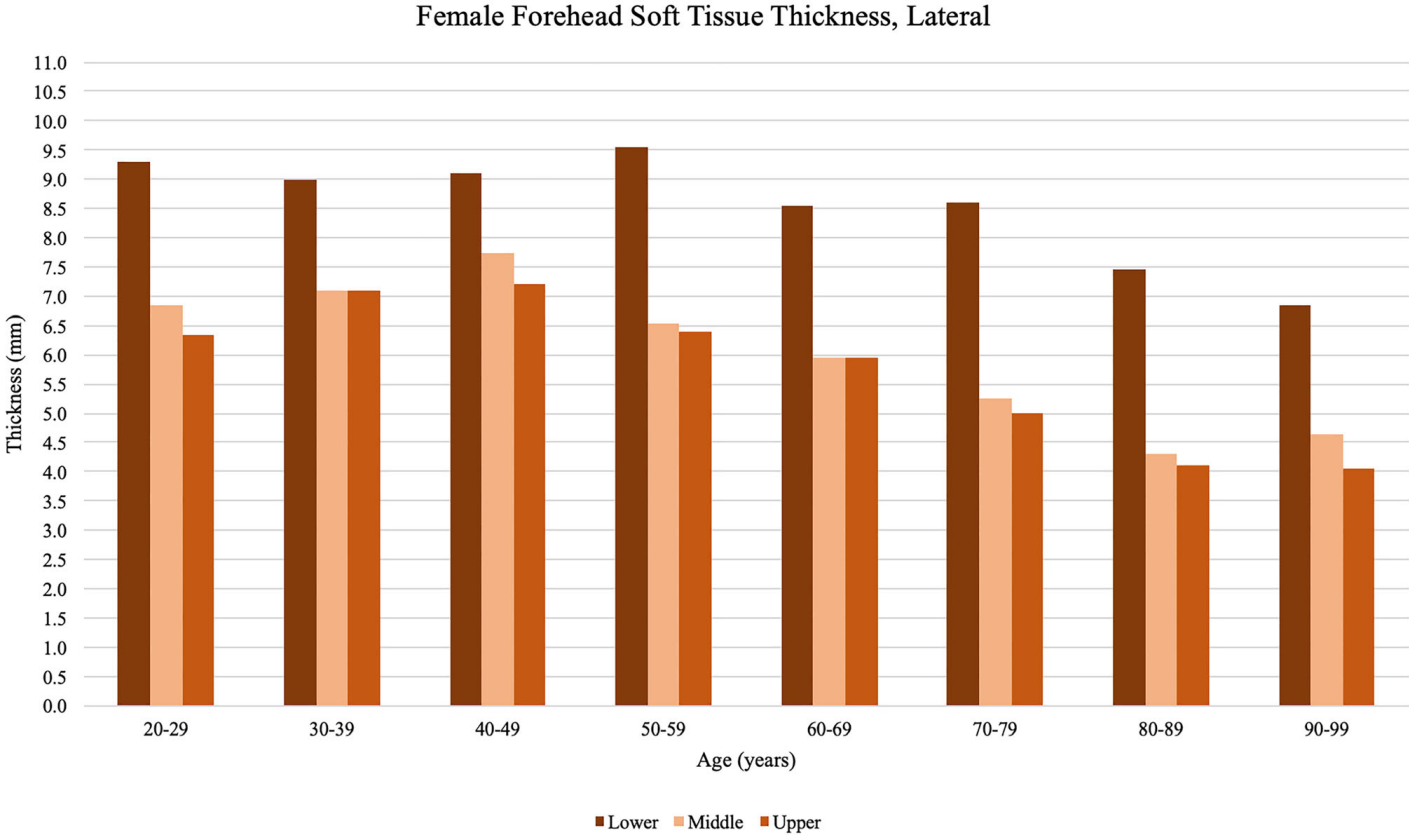
Lateral forehead soft tissue thickness measurements obtained from female participants, organized by the age of participant at the time of imaging. Peak thickness is reached by the fifth decade of life and subsequently decreases with age. At the lateral forehead, females showed thicker measurements at the lower third compared to the middle and upper thirds

Differences in forehead soft tissue by age are shown in Table 2 and Figs. 3, 4, 5, and 6. Soft tissue thickness varied significantly by participant age for all forehead locations measured in this study. Average forehead soft tissue thickness increased for all forehead locations in consecutive decades between ages 20–29 to 30–39, and 30–39 to 40–49. Average forehead soft tissue thickness then consistently decreased for all forehead locations with an eventual nadir in the ninth or tenth decade. Soft tissue was consistently thicker in the lower thirds compared to the middle and upper thirds at both the midline and lateral locations. Soft tissue was consistently thickest in the lower third of the lateral forehead at all age groups. Finally, soft tissue was consistently thinnest in the upper third of the midline forehead at all age groups.

**Table 2:** Average forehead soft tissue measurements at each location organized by age of participant

Forehead location		Age of participant at time of imaging								
		20–29	30–39	40–49	50–59	60–69	70–79	80–89	90–99	*p*-value
Midline	Upper 1/3rd	4.8	5.7	5.9	5.1	4.9	4.8	3.8	3.7	**<0.001**
	Middle 1/3rd	5.2	5.9	6.3	5.2	5.1	5.2	4.1	4.5	**<0.001**
	Lower 1/3rd	6.0	7.0	7.7	6.4	6.7	6.3	5.8	5.7	**0.007**
	Average	5.3	6.2	6.6	5.5	5.5	5.4	4.6	4.6	**<0.001**
Lateral	Upper 1/3rd	6.5	6.8	7.5	6.3	6.4	5.5	4.7	4.7	**<0.001**
	Middle 1/3rd	6.5	7.0	7.7	6.6	6.6	5.5	4.8	5.4	**<0.001**
	Lower 1/3rd	9.2	9.2	9.9	9.7	9.0	8.7	8.2	7.6	**0.004**
	Average	7.4	7.8	8.4	7.6	7.3	6.6	5.9	5.9	**<0.001**

Bold vaues indicate *p* < 0.05

Thickness measurements reported in millimeters

## Discussion

While prior research has shown differences in soft tissue thickness between genders and across age groups in various anatomical locations, comparing findings across these studies is challenging due to inconsistencies in measurement techniques, study population, and specific anatomical locations investigated. Different modalities described in the literature to measure skin thickness for analysis include ultrasonography, optical coherence tomography (OCT), confocal laser scanning microscopy (CLSM), structured-light 3D scanners, manual calipers, and skin biopsies, among others [6–25]. While biopsy is often touted as the gold standard for examining tissue layers, the preparation and processing of tissue can introduce error when measuring tissue thickness and may be less accurate than methods such as MRI. Cadaver biopsies specifically likely fail to accurately represent living tissue [9, 33, 34]. Ultrasound has also been a popular choice for similar studies in the past but is highly operator dependent. A study by Ernst et al. demonstrated that, using MRI as a control, the amount of compression by the ultrasound operator can significantly affect the accuracy of soft tissue thickness measurements [37]. Another study by Kozáková et al. found that ultrasound measurements of facial soft tissue are highly dependent on position of the subject (i.e., upright vs. supine) due to the effects of gravity [38]. By using MRI, we hope to avoid these potential confounders.

A common limitation found throughout our review of the literature was grouping of the subjects into broad age cohorts, often with all patients over 50–60 in one group [11, 24, 27, 30–32]. Others fail to include patients in their 60s and older [8, 18]. By using a large sample size and grouping cohorts by decade, we were able to identify more granular, age-related trends across the entire adult lifespan. Forehead soft tissue thickness for both males and females in this study peaked in the fifth decade of life. Thickness subsequently declined, with a marked reduction to its lowest point in the 80–89 and 90–99 age groups. This pattern of increased thickness from youth to middle age, followed by a decline, adds clarity to the conflicting reports in the existing literature with some studies finding increases and other studies reporting decreases in forehead skin thickness with advancing age [11, 26, 39, 40]. Skin undergoes a period of early growth due to dermal collagen rearrangement and accumulation of elastin proteins [3, 11, 26, 41, 42]. Our study's findings support the idea that cumulative intrinsic and extrinsic processes lead to progressive skin forehead thinning with age. As individuals enter middle age, natural intrinsic degradation processes occur, including reduced collagen synthesis, elastin fragmentation, dehydration, decreased water retention, epidermal papillae retraction, lipid loss in the stratum corneum, and flattening of the dermal–epidermal junction with reduced rete peg interdigitation [7, 15, 16, 43]. External photoaging and shear-induced mechanical damage further contribute to epidermal thinning and decreased overall skin thickness.

Males in this study generally exhibited greater forehead soft tissue thickness compared to females for all forehead locations, especially laterally. Review of the literature documents inconsistency in data comparing forehead skin thickness by gender. Some studies have reported greater overall thickness in male forehead skin compared to that of females [18, 27]. This may be attributed to more robust collagen density and higher collagen density in males, as well as post-menopausal estrogen declines in females [1, 10, 24, 44–46]. Multiple studies using optical coherence tomography imaging reported no gender-related differences in epidermal thickness between younger males and females, but significantly thicker forehead epidermal thickness in older males than in older females [8, 24]. Although gender seems to be associated with forehead skin thickness, these findings suggest that age may be a stronger predictor of skin thickness.

Changes in forehead thickness also varied by specific forehead location. In this study, lateral forehead soft tissue thickness was consistently thicker than that of the medial forehead for all corresponding thirds. This is inconsistent with several studies that reported thinner lateral forehead skin than central skin. It is important to note, however, that these studies performed their lateral forehead measurements at the temple region, lateral to the temporal ridge. Our study, on the other hand, performed lateral forehead measurements at the level of the supraorbital foramen, medial to the temporal ridge. Our study also measured total soft tissue thickness from epidermis to bone (i.e., including subcutaneous fat, muscle, and pericranium). These other studies focused on skin thickness alone, which may explain the notably different findings upon comparison of medial and lateral forehead regions. In this study, thickness in the lower forehead was greater than the upper forehead in both lateral and midline positions. This is consistent with prior studies that reported greater forehead skin thickness in the lower forehead skin compared to upper forehead skin [28]. These findings may be attributed to several factors. Firstly, midline thinning may be associated with morphological variations in frontalis decussation, as well as differences in midline movement and structural support [47–49]. Imbalanced lateral forehead/brow attenuation, dependent edema, and differences in corrugator and frontalis muscle thickness might also contribute to these findings. Regardless of the etiology, these data suggest that the lower third of the lateral forehead may be more resistant to age-related soft tissue thinning than the upper and middle thirds of the lateral forehead.

Age- and gender-related variations in forehead soft tissue thickness have clinical significance for clinicians seeking to address facial aging and plan appropriate esthetic interventions. The forehead is a critical area in esthetic medicine that is often the target of anti-aging treatments. This study revealed consistent regional variability in forehead soft tissue thickness that should help to refine esthetic treatment strategies. Thicker soft tissue in the lower third likely minimizes the risk of visible irregularities with volume restoration techniques such as dermal fillers and fat grafting. Similarly, thicker soft tissue in this region may better tolerate deeper and more aggressive treatments with microneedling and microcoring devices, as well as ultrasound and radiofrequency devices that rely on energy delivery to precise tissue depths. Thinner soft tissue areas, such as those in the middle and upper thirds of the midline, might be better treated with more conservative volume restoration to avoid visible irregularities, as well as less aggressive laser energy settings and lighter peeling techniques to ensure that treatments balance effectiveness while minimizing the risk of untoward effects such as hypopigmentation, scarring, or burns [50–53]. Our findings also support the importance of taking age and gender into consideration when performing laser, chemical peel, and other resurfacing techniques. As the forehead thins with age, particularly after the fifth decade of life, the skin becomes more susceptible to the effects of extrinsic factors such as gravity, mechanical stress, and photodamage. Younger patients with thicker tissue may tolerate more aggressive treatments, while older patients with thinner tissue may require more conservative approaches to minimize complications. Similarly, male patients may necessitate more aggressive treatment options to address thicker forehead soft tissue than female counterparts. In any case, understanding the effects of aging and gender on forehead soft tissue thickness gives providers the opportunity to plan more nuanced surgical procedures of the forehead and brow. Consideration of the anatomical and demographic factors presented in this study should help esthetic providers to tailor treatments, optimize outcomes, and improve patient satisfaction [54, 55]. Finally, there is notable applicability of this anatomical data to other fields of plastic surgery, such as planning for reconstructive procedures of the forehead, eyebrows, and nose.

Limitations of this study are largely related to data collection and patient cohort characteristics. MRI imaging appeared to be an accurate and reliable tool for measuring forehead soft tissue thickness in this study. It is a non-invasive modality that proved particularly advantageous for analyzing larger retrospective datasets. Furthermore, it provides superior contrast resolution to ultrasound, allowing for clear visualization of subcutaneous structures and differentiation between various soft tissue layers. The use of reproducible boney landmarks allows for more consistently objective thickness measurements that can be implemented and standardized for future studies. That being said, MRI measurements can be inherently flawed by user measurement subjectivity, though arguably less so than ultrasound. Our data were collected by one individual to maintain consistency, but this could introduce bias. Future studies using similar methods could employ multiple researchers to take measurements with statistics performed to ensure consistency between observers. Another significant limitation of this study lies in measurement of total forehead soft tissue thickness as a soft tissue composite rather than specifically examining the individual skin layers (e.g., epidermal thickness, dermal thickness, and subcutaneous fat thickness). Measuring the total soft tissue thickness limits the granularity, specificity, and generalizability of the findings. Of note, this composite thickness approach was most appropriate with MRI imaging. Nevertheless, it inherently overlooks differences in skin layers that likely play a critical role in aging- and gender-related changes. In a similar sense, composite thickness measurement likely overestimates changes in muscle and pericranium thickness, especially upon comparison to prior studies. It is, therefore, difficult to infer conclusions specifically about forehead skin using results from this study. Our study also does not control for BMI. There is limited literature examining the relationship between body mass index (BMI) and facial skin thickness. Some small studies suggest a positive correlation, indicating that higher BMI may be associated with increased facial skin thickness [29, 31]. However, other research has found no significant effect of BMI on facial skin thickness [30]. This discrepancy highlights a gap in our understanding and presents a valuable opportunity for future studies to explore this relationship in greater depth. Finally, the literature has shown epidermal and dermal skin thickness to correlate with increased Fitzpatrick skin type, which was not taken into account in the current study [16]. Only Caucasian patients were included in this review, which is certainly an opportunity for study expansion.

## Conclusion

This study presents a unique evaluation of forehead soft tissue thickness with a large sample size across a broad age range via consistent anatomical landmarks. MRI data from this study suggest that forehead soft tissue thickness varies significantly by age and gender. Men generally have thicker forehead soft tissue, and both genders generally experience a peak in thickness in midlife followed by a subsequent decline. By integrating these findings into clinical practice, providers can ultimately improve patient outcomes of esthetic forehead procedures. Future studies which use MRI to measure soft tissue thickness stratified by race, ethnicity, BMI, and Fitzpatrick skin type would be beneficial additions to the literature.

## Abbreviation

MRI: Magnetic resonance imaging
